# Multidrug resistant bacterial infection in liver transplant recipients: A Deterrent and obstacle

**DOI:** 10.12669/pjms.42.8.15347

**Published:** 2026-08

**Authors:** Nazish Misbah, Jahanzaib Haider, Muhammad Hassan, Mehreen Fatima

**Affiliations:** 1 Nazish Misbah, FCPS (Medicine), FCPS (Infectious Disease), Dow University Hospital, Dow University of Health Sciences, Karachi Pakistan; 2 Jahanzaib Haider, MCPS, MHPE, FCPS, Fellowship in Liver Transplant and HPB Surgery Liver Transplant and Hepatopancreatobiliary Surgery Unit, Dow University Hospital, Dow University of Health Sciences, Karachi Pakistan; 3 Muhammad Hassan, FCPS, Fellowship in Liver Transplant and HPB Surgery Liver Transplant and Hepatopancreatobiliary Surgery Unit, Dow University Hospital, Dow University of Health Sciences, Karachi Pakistan; 4 Mehreen Fatima, FCPS (Medicine), FCPS (Infectious Disease) Department of Infectious Disease, Dow University Hospital, Dow University of Health Sciences, Karachi Pakistan

**Keywords:** Antimicrobial Stewardship, Bacterial, Carbapenem Resistance, Catheter-Related Infections, Cross Infection, Drug Resistance, Intraabdominal Infections, Liver Transplantation, Multiple, Risk Factors, Sepsis

## Abstract

**Objectives:**

To determine the microbiological spectrum of multi-drug resistant (MDR) bacterial infections within the first 30 days post-living donor liver transplantation (LDLT) and identify the associated risk factors contributing to their emergence.

**Methodology:**

This retrospective cohort study reviewed the medical records of patients who underwent LDLT from January 2020 to December 2024 at the Liver Transplant and Hepatopancreatobiliary Surgery Unit of Dow University Hospital, Karachi. Patients were categorized into MDR and no-MDR groups based on culture-confirmed MDR bacterial infection with clinical features of sepsis. Types of MDR bacterial infections were noted. Baseline, pre-transplant, intraoperative, and post-transplant variables were recorded. Pearson Chi-square test, Mann-Whitney U test, and univariate and multivariate logistic regression analyses were performed for statistical inference.

**Results:**

Of 151 LDLT recipients, 75 (49.7%) developed MDR bacterial infections within 30 days. Among these, gram negative MDR organisms were predominated, with carbapenem-resistant Enterobacteriaceae (21.2%) and carbapenem-resistant *Acinetobacter baumannii* (20.5%) most frequent, followed by vancomycin-resistant *Enterococci* (13.2%). On univariate analysis, MDR infection was associated with pre-transplant ICU admission, renal dysfunction, prolonged postoperative ICU stay, mechanical ventilation >48 h, hemodialysis and/or sustained low-efficiency dialysis (SLED), postoperative complications, longer invasive device duration, prolonged hospital stay, central line-associated blood stream infection (CLABSI), and intra-abdominal infections. On multivariate analysis, only CLABSI [Wald χ²=17.696 (OR 0.021; 95% CI: 0.003–0.127; p<0.001)] and intra-abdominal infections [Wald χ²=25.567 (OR 0.008; 95% CI: 0.001–0.050; p<0.001)] remained independently associated with MDR infection.

**Conclusion:**

Nearly half of LDLT recipients developed MDR infections, predominantly due to gram-negative organisms. CLABSI and intra-abdominal infections emerged as the key predictors of MDR bacterial infections.

## INTRODUCTION

Liver transplantation (LT) continues to serve as the cornerstone of treatment for patients suffering from advanced stage liver disease and early primary liver tumors, offering both the possibility of a cure and enhanced long-term survival.^1^-^3^ Over the years, surgical refinements and better perioperative care have markedly improved patient outcomes;^3^ however, infections in the early postoperative period remain a serious concern due to their association with high mortality rates.^4^-^6^ Among various causes, bacterial infections are the most frequently encountered complications within the first 30-days after LT.^7^,^8^

A number of risk factors are present in LT recipients for colonization and infections by multi-drug resistant (MDR) organisms. These include immune dysfunction related to chronic liver disease, extended hospital and ICU stays, and exposure to broad-spectrum antibiotics in the preoperative settings.^9^-^11^ The risk is further amplified in the postoperative period due to the increased translocation of bacteria from the gut and peritoneal cavity into the bloodstream and the institution of immunosuppressive regimen.^12^-^14^ Therefore, managing these MDR infections is complicated by limited treatment options and the frequent ineffectiveness of empirical antibiotic therapies. This issue is particularly distinct in resource-limited settings like Pakistan, where access to new antimicrobial drugs is restricted.^15^,^16^

Common MDR bacterial infections involved in early post-transplant infections include extended-spectrum β-lactamase (ESBL) producing Enterobacteriaceae (*Escherichia coli* and *Klebsiella species*), gram-negative bacteria such as *Pseudomonas aeruginosa* and *Acinetobacter baumannii*, and gram-positive bacteria such as Methicillin-resistant *Staphylococcus aureus* (MRSA) and vancomycin-resistant *enterococci* (VRE).^7^,^8^,^17^ Although MDR infections are prevalent among immunocompromised individuals worldwide,^7^,^8^,^10^,^11^ there is a notable lack of published research from various liver transplant centers in Pakistan that specifically addresses the patterns and risk factors associated with MDR infections in the context of LT. Therefore, this study examined the microbiological spectrum of MDR bacterial infections within the first 30 days post-living donor liver transplantation (LDLT) and identified the associated risk factors contributing to their emergence.

## METHODOLOGY

This retrospective cohort study reviewed the medical records of patients who underwent LDLT from January 2020 to December 2024 at the Liver Transplant and Hepatopancreatobiliary (HPB) Surgery Unit of Dow University Hospital, Ojha Campus, Dow University of Health Sciences (DUHS), Karachi, Pakistan. A non-probability consecutive sampling technique was employed, whereby all eligible LDLT recipients during the study period were included.

### Inclusion and exclusion criteria:

Patients were grouped based on the presence or absence of MDR bacterial infections (MDR group vs. no MDR group, respectively) based on microbiological culture reports along with clinical parameters (i.e., occurrence of systemic inflammatory response syndrome^18^). Patients with viral, fungal, or parasitic infections were excluded from the study.

### Ethical approval:

An exemption from the Institutional Review Board (IRB) of the DUHS was granted (Approval No. IRB-3886/DUHS/EXEMPTION/2025/59, Dated: February 20, 2025).

### Operational definitions:

A bacterial isolate resistant to at least one antimicrobial agent in three or more antibiotic classes was defined as an MDR bacterial infection. The antimicrobial categories used for this classification included carbapenems (meropenem, imipenem-cilastatin), extended-spectrum penicillins with beta-lactamase inhibitors (piperacillin-tazobactam), fluoroquinolones (ciprofloxacin, levofloxacin), aminoglycosides (gentamicin, amikacin, tobramycin), and third- and fourth-generation cephalosporins for gram-negative organisms. For gram-positive organisms, resistance to vancomycin was used to define VRE, and resistance to methicillin was used to define MRSA, as reported on individual culture and sensitivity (CS) reports in accordance with the Clinical and Laboratory Standards Institute (CLSI) M100 breakpoints applicable during the study period.^19^ Spontaneous bacterial peritonitis (SBP) was defined as ≥250 polymorphonuclear leukocytes per mm³ of ascitic fluid, and an increase in serum creatinine of ≥0.3 mg/dL within 48 h was defined as renal dysfunction.

### Data collection:

A broad range of clinical variables was divided into four categories: baseline, pre-transplant, intraoperative, and postoperative.

### Baseline data:

Included patient age group, sex, body mass index (BMI), blood group, comorbidities such as diabetes mellitus and hypertension, primary liver disease, presence of hepatocellular carcinoma, Child-Turcotte-Pugh (CTP), Model for End-Stage Liver Disease and sodium (MELD-Na) scores. Pre-transplant variables included the preceding three months and any previous MDR isolate, rifaxamin use, hospitalization, and/or ICU admission. Other factors included donor infection (blood or urine), previous history of ascites, SBP, variceal bleeding and/or portosystemic encephalopathy (PSE), and pre-transplant renal dysfunction. The intraoperative parameters included surgery duration (>8 h), cold and warm ischemia times (min), blood loss (mL), transfusion requirements (>10 units of blood products), and biliary anastomosis (duct-to-duct anastomosis or hepaticojejunostomy). Postoperative records included ICU stay length (>7 days), need for mechanical ventilation (>48 h), hemodialysis and/or sustained low-efficiency dialysis (SLED), and complications such as reactionary bleeding, abdominal collections, high drain output >1000 mL/day), pleural effusion, acute cellular rejection (requiring pulse therapy), re-exploration, and/or vascular events (hepatic artery and/or portal vein thrombosis). The duration of indwelling device use (days), including the Foley catheter, central venous pressure (CVP), arterial lines, and abdominal drains, was documented along with the total hospital stay (days).

### Microbiological profile:

Microbiological data were analyzed to determine the infection source. Culture-confirmed conditions, such as ventilator-associated pneumonia (VAP), central line-associated bloodstream infection (CLABSI), catheter-associated urinary tract infection (CAUTI), and intra-abdominal infections (positive CS of drain fluid and/or abdominal collections), were included based on combined clinical and microbiological criteria. These parameters were also considered additional risk factors for the occurrence of MDR bacterial organisms and analyzed as non-mutually exclusive events, as a single patient could develop infections at multiple anatomical sites during the same admission. The types of MDR bacterial infections were also noted.

### Specimen collection:

Microbiological specimens were collected following standardized institutional protocols. Two sets of aerobic and anaerobic blood cultures were drawn simultaneously (one from a peripheral vein and one through the central venous catheter) when CLABSI was clinically suspected. Midstream clean-catch or catheter-obtained urine specimens were submitted for culture in cases of suspected CAUTI. For patients on mechanical ventilation, endotracheal aspirates were collected when VAP was suspected. Drain fluid and/or aspirates from abdominal collections were sent for culture to evaluate intra-abdominal infections. All specimens were processed in the hospital’s microbiology laboratory using standard culture methods, with organism identification and antimicrobial susceptibility testing performed by disc diffusion and/or minimum inhibitory concentration (MIC) determination.

### Retrospective evaluation of culture reports:

All culture and sensitivity reports generated within 30 days of LDLT were retrieved from the hospital’s laboratory records and cross-referenced with the corresponding medical records. Reports were reviewed in chronological order and screened for organism identification and antimicrobial resistance patterns to minimized information bias. Cases were classified as MDR only when resistance to at least one antimicrobial agent in three or more antibiotic classes and clinical evidence of infection (meeting SIRS criteria) were concurrently satisfied. When a patient had multiple culture results, each isolate was evaluated independently by the corresponding author; however, cases were counted once per patient per site of infection to avoid double-counting. As a single patient could develop infections at more than one site during the same admission, patients may therefore contribute to more than one infection category, and these events were analyzed as non-mutually exclusive. Selection bias was inherent to the retrospective, single-center design; however, consecutive inclusion of all eligible LDLT recipients during the study period minimized systematic exclusion.

### Statistical analyses:

All analyses were performed using SPSS version 23. Categorical data were summarized as frequencies and percentages, and continuous data were described as medians and interquartile ranges (IQRs). Comparisons between the two groups were made using the Pearson Chi-square test for categorical variables and the Mann–Whitney U test for continuous variables. Univariate binary logistic regression was performed to estimate the odds ratios (ORs) and 95% confidence intervals (CIs) for the potential predictors of MDR infection. Variables with a p-value <0.10 in the univariate analysis were included in a multivariate logistic regression model to identify independent risk factors. Statistical significance was set at p <0.05.

## RESULTS

### Baseline Characteristics:

Of the 151 LDLT recipients included in this study, 75 (49.7%) had MDR bacterial infections: 56 (74.7%) males and 19 (25.3%) females, with a median age of 45 years (IQR: 38-54). In the no MDR group, 61 (80.3%) were males and 15 (19.7%) were females with a median age of 45 years (IQR: 36-53). No differences were observed in BMI, primary liver disease, and MELD-Na score between the two groups. Although CTP class C was predominant in both groups [47 (62.7%) patients in the MDR group vs. 47 (61.8%) patients in the no MDR group], the results were not statistically significant (p=0.991). The additional baseline characteristics are summarized in Table-I.

**Table-I:** Baseline Characteristics.

Variables	MDR group n=75 (49.7%)	No MDR group n=76 (50.3%)	Test-statistics	p-value
Age (years) [Median (IQR)]	45 (38-54)	45 (36-53)	U=2765.0	0.858
***Sex [n (%)]***			χ²=0.678	0.410
•Male	56 (74.7)	61 (80.3)		
•Female	19 (25.3)	15 (19.7)		
^a^BMI (in kg/m^2^) [Median (IQR)]	24.3 (21.6-29)	24.7 (21.5-28)	U=2555.5	0.573
Diabetes Mellitus [n (%)]	24 (32)	17 (22.4)	χ²=1.770	0.183
Hypertension [n (%)]	09 (12)	05 (6.6)	χ²=1.319	0.251
***Ethnicity [n (%)]***			χ²=0.382	0.536
•Urban residence	50 (66.7)	47 (61.8)		
•Rural residence	25 (33.3)	29 (38.2)
***Blood group [n (%)]***			χ²=2.441	0.486
•Blood group A	20 (26.7)	26 (34.2)		
•Blood group B	21 (28)	22 (28.9)		
•Blood group O	27 (36)	19 (25)		
•Blood group AB	07 (9.3)	09 (11.8)
***Primary liver disease [n (%)]***			χ²=3.947	0.684
•HBV	22 (29.3)	24 (31.6)		
•HCV	27 (36)	31 (40.8)		
•Alcoholic liver disease	02 (2.7)	02 (2.6)		
^•b^NASH	03 (04)	0 (0)		
•Wilson disease	01 (1.3)	02 (2.6)		
•Autoimmune hepatitis	01 (1.3)	01 (1.3)		
•Others	19 (25.3)	16 (21.1)		
^c^MELD-Na score [Median (IQR)]	18 (13-22)	16 (12.5-22)	U=2643.0	0.440
***^d^CTP score [n (%)]***			χ²=0.018	0.991
•Grade A (5-6)	08 (10.7)	08 (10.5)		
•Grade B (7-9)	20 (26.7)	21 (27.6)		
•Grade C (>10)	47 (62.7)	47 (61.8)		
Hepatocellular carcinoma (HCC) [n (%)]	23 (30.7)	31 (40.8)	χ²=1.684	0.194

a=Body mass index; b=Non-alcoholic steatohepatitis; c=Model for end stage liver disease and sodium;

d=Child-Pugh Turcotte; χ²=Pearson Chi-square test; U=Mann-Whitney U test.

### Pre-transplant, intraoperative, and postoperative factors:

The pre-transplant, intraoperative, and postoperative risk factors are shown in Table-II.

**Table-II:** Pre-transplant, intraoperative and postoperative risk factors.

Variables	MDR group n=75 (49.7%)	No MDR group n=76 (50.3%)	Test Statistics	p-value
***Pre-transplant risk factors***				
Isolation of ^a^MDR (last 03-months pre-transplant) [n (%)]	13 (17.3)	08 (10.5)	χ²=1.461	0.227
Donor infection status (blood/urine) [n (%)]	01 (1.3)	02 (2.6)	χ²=0.327	0.568
Rifaxamin exposure (0-3 months pre-transplant) [n (%)]	38 (50.7)	40 (52.6)	χ²=5.361	0.147
Hospitalization (0-3 months pre-transplant) [n (%)]	36 (48)	25 (32.9)	χ²=3.577	0.059
^b^ICU admission (0-3 months pre-transplant) [n (%)]	15 (20)	06 (7.9)	χ²=4.620	0.032
Previous history of ascites [n (%)]	58 (77.3)	60 (78.9)	χ²=0.058	0.810
Previous history of ^c^SBP [n (%)]	06 (08)	05 (06)	χ²=0.113	0.737
Previous history of variceal bleed [n (%)]	21 (28)	23 (30.3)	χ²=0.094	0.760
Previous history of ^d^PSE [n (%)]	30 (40)	34 (44.7)	χ²=0.347	0.556
Previous renal dysfunction [n (%)]	13 (17.3)	05 (6.8)	χ²=3.806	0.051
***Intraoperative risk factors***				
Duration of surgery (>8 hours) [n (%)]	51 (68)	47 (61.8)	χ²=0.628	0.428
Cold ischemia time (minutes) [Median (IQR)]	40 (33-50.5)	40 (28-51)	U=2398.5	0.436
Warm ischemia time (minutes) [Median (IQR)]	40 (33.5-48.5)	42 (33-50)	U=2480.5	0.656
Blood loss (mls) [Median (IQR)]	800 (550-1500)	800 (500-1500)	U=2526.0	0.587
Transfusion requirement (>10 units blood products) [n (%)]	22 (29.3)	24 (31.6)	χ²=0.094	0.760
***Biliary anastomosis [n (%)]***				
•Duct-to-duct anastomosis	67 (89.3)	70 (92.1)	χ²=0.345	0.557
•Hepaticojejunostomy	08 (10.7)	06 (7.9)
***Postoperative risk factors***				
ICU stay (>7 days) [n (%)]	28 (37.3)	18 (23.7)	χ²=3.320	0.068
Mechanical ventilation (>48 h) [n (%)]	31 (41.3)	19 (25)	χ²=4.547	0.033
Hemodialysis and/or ^e^SLED [n (%)]	16 (21.3)	02 (2.6)	χ²=12.575	<0.001
***Complications [n (%)]***				
•Reactionary bleeding	08 (10.7)	07 (9.2)	χ²=0.089	0.765
•Abdominal collections	24 (32)	11 (14.5)	χ²=6.512	0.011
•High drain output	16 (21.3)	10 (13.2)	χ²=1.770	0.183
•Pleural effusion	26 (34.7)	10 (13.2)	χ²=9.618	0.002
•Acute rejection (requiring pulse therapy)	03 (4.1)	02 (2.9)	χ²=0.141	0.707
•Re-exploration	08 (10.7)	03 (3.9)	χ²=2.523	0.112
•Vascular events	13 (17.3)	05 (6.6)	χ²=4.158	0.041
Duration of Foleys catheter (days) [Median (IQR)]	09 (6-12)	06 (4.7-9)	U=1711.0	<0.001
Duration of ^f^CVP line (days) [Median (IQR)]	05 (4-6.5)	04 (3-5)	U=2017.0	0.010
Duration of arterial line (days) [Median (IQR)]	04 (3-5)	03 (2.7-4)	U=2008.5	0.011
Duration of abdominal drains (days) [Median (IQR)]	13 (10-15)	10 (8-13)	U=1497.0	<0.001
Duration of hospital stay (days) [Median (IQR)]	14 (10.5-19)	11 (9-13)	U=1506.5	<0.001

a=Multi-drug resistant; b=Intensive care unit; c=Spontaneous bacterial peritonitis; d=Portosystemic encephalopathy; e=Sustained low-efficiency dialysis; f=Central venous pressure; χ²=Pearson Chi-square test; U=Mann-Whitney U test.

### Pre-transplant risk factors:

Within the three months preceding LDLT, 21 (13.9%) patients had an MDR isolate; however, prior MDR isolation did not significantly predict post-transplant MDR infection [13 patients (17.3%) in the MDR group compared to 08 patients (10.5%) in the no MDR group; p=0.227]. Similarly, prior donor infection, rifaxamin exposure, prior hospitalization, and pre-transplant renal dysfunction did not show statistically significant differences between the groups. There were significantly higher MDR infection rates in patients with prior ICU admission [15 (20%) vs. 06 (7.9%); p=0.032].

### Intraoperative risk factors:

Prolonged operative time (>8 hours) was encountered in 98 (64.9%) cases and was not significantly associated with MDR infection [51 (68%) in the MDR group vs. 47 (61.8%) in the no MDR group; p=0.428]. Correspondingly, intraoperative blood transfusion requirements and ischemia times (cold and warm) showed no significant relationship with post-transplant MDR occurrence.

### Postoperative risk factors:

Several postoperative variables were significantly associated with MDR infection. The postoperative need for mechanical ventilation (>48 h) and institution of hemodialysis and/or SLED were significantly associated with MDR infection (p=0.033 and p<0.001, respectively). Likewise, the presence of abdominal collections (p=0.011), pleural effusion (p=0.002), and vascular events (p=0.041) were also significantly associated with post-transplant MDR infections. The median (IQR) duration of invasive device use was significantly longer among MDR patients, including Foley catheter [09 (6-12) vs. 06 (4.7-9) days; p < 0.001], central venous catheter [05 (4-6.5) vs. 04 (3-5) days; p = 0.010], arterial line [04 (3-5) vs. 03 (2.7-4) days; p = 0.011], and abdominal drains [13 (10-15) vs. 10 (8-13) days; p < 0.001]. Patients with MDR infections also experienced longer median (IQR) duration of hospital stay [14 (10.5-19) days vs.11 (9-13) days; p<0.001].

### Microbiological profile:

**Fig.1:**
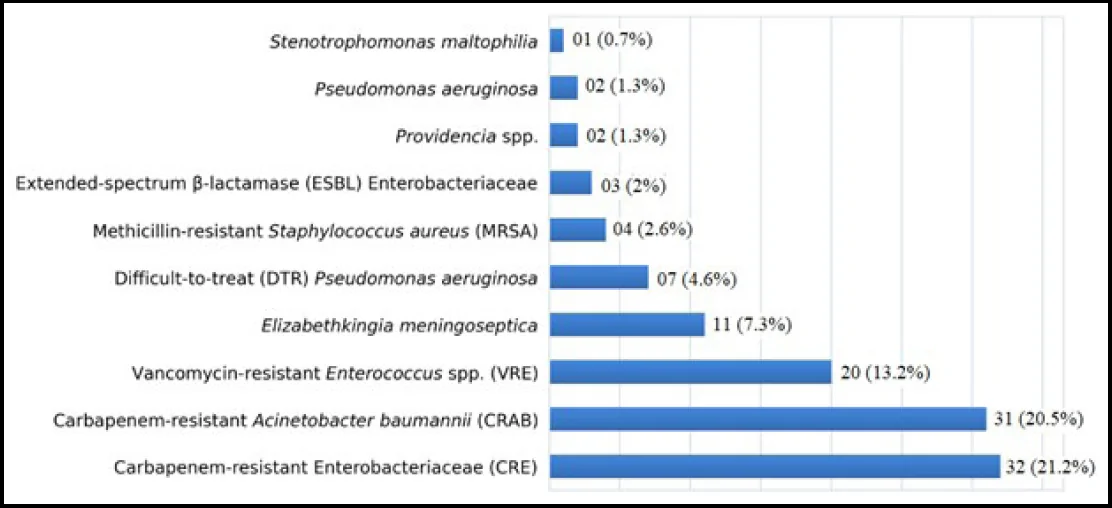
Types of MDR bacterial organisms (n=151).

CLABSI was markedly more frequent in the MDR group than in the no MDR group [32 (42.7%) vs. 06 (7.9%), p<0.001)]. Similarly, intra-abdominal infections were statistically significant in the MDR group compared to the no MDR group [43 (57.3%) vs. 03 (3.9%), p<0.001)] (Table-III). Among all post LDLT - MDR infections, the most prevalent pathogens were carbapenem-resistant Enterobacteriaceae (CRE) in 32 (21.2%), carbapenem-resistant *Acinetobacter baumannii* (CRAB) in 31 (20.5%), and VRE in 20 (13.2%), followed by *Elizabethkingia meningoseptica* in 11 (7.3%) and difficult-to-treat (DTR) *Pseudomonas aeruginosa* in 07 (4.6%) patients (Fig.1).

**Table-III:** Sources of MDR bacterial organisms.

Variables	MDR group n=75 (49.7%)	No MDR group n=76 (50.3%)	Test Statistics	p-value
^a^VAP [n (%)]	11 (14.7)	09 (11.8)	χ²=0.262	0.609
^b^CLABSI [n (%)]	32 (42.7)	06 (7.9)	χ²=22.004	<0.001
^c^CAUTI [n (%)]	02 (2.7)	01 (1.3)	χ²=0.454	0.500
Intra-abdominal infections (positive drain fluid and/or abdominal collection ^d^CS) [n (%)]	43 (57.3)	03 (3.9)	χ²=50.788	<0.001

Percentages are calculated using the total number of patients in each group as the denominator. Individual patients could have more than one site of infection; therefore, cumulative frequencies and percentages may not equal the total number of patients in each group.

a=Ventilator-associated pneumonia; b=Central line-associated bloodstream infection; c=Catheter-associated urinary tract infection; d=Culture and sensitivity; χ²=Pearson Chi-square test.

### Predictors of MDR bacterial infections:

In univariate logistic regression analysis, several variables were significantly associated with MDR infection, including pre-transplant hospitalization, pre-transplant ICU admission, renal dysfunction, post-transplant ICU stay, mechanical ventilation, hemodialysis/SLED, post-transplant complications (such as abdominal collections, pleural effusion, and vascular events), prolonged Foley, CVP and arterial lines, abdominal drain durations, prolonged hospitalization, CLABSI, and intra-abdominal infections. In the multivariate model, two factors remained independently associated with MDR infection. CLABSI significantly increased the risk of MDR infection [Wald χ²=17.696 (OR 0.021, 95%CI: 0.003-0.127; p<0.001)], and intra-abdominal infections were also independently associated with MDR infection [Wald χ²=25.567 (OR 0.008, 95%CI: 0.001-0.050; p<0.001)]. Other variables lost their significance after adjusting for the confounders (Table-IV).

**Table-IV:** Univariate and multivariate analyses of predictors of MDR bacterial organisms.

Risk factors of ^a^MDR	MDR group n=75	No MDR group n=76	Univariate [OR (95% CI)]	Wald χ²	p-value	Multivariate [OR (95% CI)]	Wald χ²	p-value
Hospitalization (0-3 months pre-transplant) [n (%)]	36 (48)	25 (32.9)	0.531 (0.275-1.026)	3.544	0.060	0.655 (0.152-2.814)	0.324	0.569
^b^ICU admission (0-3 months pre-transplant) [n (%)]	15 (20)	06 (7.9)	0.343 (0.125-0.939)	4.336	0.037	0.384 (0.044-3.387)	0.742	0.389
Pre-transplant renal dysfunction [n (%)]	13 (17.3)	05 (6.8)	0.351 (0.118-1.040)	3.568	0.059	1.592 (0.182-13.903)	0.177	0.674
ICU stay (>7 days) [n (%)]	28 (37.3)	18 (23.7)	0.521 (0.257-1.055)	3.277	0.070	4.284 (0.661-27.781)	2.327	0.127
Mechanical ventilation (>48 h) [n (%)]	31 (41.3)	19 (25)	0.473 (0.236-0.946)	4.475	0.034	3.059 (0.520-18.001)	1.529	0.216
Hemodialysis/^c^SLED [n (%)]	16 (21.3)	02 (2.6)	0.100 (0.022-0.451)	8.968	0.003	0.065 (0.002-1.946)	2.486	0.115
Abdominal collections [n (%)]	24 (32)	11 (14.5)	0.360 (0.161-0.802)	6.242	0.012	0.751 (0.164-3.437)	0.137	0.712
Pleural effusion [n (%)]	26 (34.7)	10 (13.2)	0.268 (0.126-0.647)	9.027	0.003	0.405 (0.076-2.157)	1.122	0.290
Vascular event [n (%)]	13 (17.3)	05 (6.6)	0.336 (0.113-0.995)	3.876	0.049	0.290 (0.027-3.068)	1.057	0.304
Duration of Foley catheter (days) [Median (IQR)]	09 (6-12)	06 (4.7-9)	1.158 (1.061-1.264)	10.714	0.001	0.989 (0.740-1.320)	0.006	0.938
Duration of ^d^CVP line (days) [Median (IQR)]	05 (4-6.5)	04 (3-5)	1.214 (1.044-1.412)	6.351	0.012	1.674 (0.956-2.933)	3.249	0.071
Duration of arterial line (days) [Median (IQR)]	04 (3-5)	03 (2.7-4)	1.212 (1.015-1.447)	4.536	0.033	0.696 (0.371-1.305)	1.277	0.258
Duration of abdominal drains (days) [Median (IQR)]	13 (10-15)	10 (8-13)	1.107 (1.034-1.185)	8.527	0.003	0.933 (0.771-1.129)	0.502	0.479
Duration of hospitalization (days) [Median (IQR)]	14 (10.5-19)	11 (9-13)	1.096 (1.034-1.162)	9.429	0.002	1.022 (0.865-1.209)	0.067	0.795
^e^CLABSI [n (%)]	32 (42.7)	06 (7.9)	0.115 (0.044-0.298)	19.839	<0.001	0.021 (0.003-0.127)	17.696	<0.001
Intra-abdominal infections (positive drain fluid and/or abdominal collection ^f^CS) [n (%)]	43 (57.3)	03 (3.9)	0.031 (0.009-0.106)	30.278	<0.001	0.008 (0.001-0.050)	25.567	<0.001

Since risk factors were not mutually exclusive, individual column totals may exceed the group totals.

a=Multi-drug resistant; b=Intensive care unit; c=Sustained low-efficiency dialysis; d=Central venous pressure; e=Central line-associated bloodstream infection; f=Culture and sensitivity; Wald χ²=Wald Chi-Square Statistics

## DISCUSSION

This study demonstrated the microbiological pattern and risk factors of MDR bacterial infections within the first 30 days of patients who underwent LDLT. Gram-negative MDR organisms predominated in nearly half of the patients, particularly CRE and CRAB. Importantly, CLABSI and intra-abdominal infections were the only independent predictors of MDR infection.

The period immediately following a transplant is a vulnerable time for the development of MDR organisms. This is due to factors such as significant immunosuppression, the stress of surgery, the use of invasive devices, and the extensive application of broad-spectrum antibiotics.^20^,^21^

In our current study, we found a 49.7% rate of MDR, indicating a considerable early infectious challenge. This finding is consistent with the report by Phichaphop et al., who demonstrated MDR infection rates approaching 62% after liver transplantation.^22^ Furthermore, region-wide analyses have shown a higher prevalence of MDR infections among Asian liver transplant recipients compared with Western cohorts.^23^ This discrepancy likely reflects differences in antibiotic stewardship practices and lower community-level MDR prevalence in Western transplant populations.^24^

Among the MDR pathogens identified in this study, gram-negative organisms predominated, including CRE in 21.2% and CRAB in 20.5%, followed by VRE in 13.2% of cases. A recent systematic review of MDR organism bloodstream infections in solid organ transplant recipients confirmed that gram-negative bacteria, particularly CRE and CRAB, are among the most frequent and lethal pathogens in the early post-transplant period, with VRE also contributing substantially among gram-positive MDR organisms.^25^ Similarly, in a recent multicenter study involving liver, kidney, and heart transplant recipients between 2016 and 2021, Adelman et al. reported that MDR organisms accounted for 45.5% of bloodstream infections, with CRE and CRAB representing the predominant pathogens, particularly after liver transplantation.^26^ In addition, our study also identified difficult-to-treat (DTR) *Pseudomonas aeruginosa* and *Elizabethkingia meningoseptica* in 4.6% and 7.3% of post-LDLT patients, respectively. Although less frequent, the emergence of these opportunistic pathogens is clinically significant because of their intrinsic antimicrobial resistance and strong association with severe infection and high mortality in immunocompromised hosts.^27^,^28^

Several studies have reported varying risk factors associated with MDR bacterial infections following liver transplantation. These variations are likely indicative of differences in patient demographics, perioperative care approaches, and infection prevention methods at various transplant centers. Hao et al. found that prolonged mechanical ventilation, ICU stays, and hospitalizations were significantly related to MDR bacterial infections in their retrospective analysis involving 350 liver transplant recipients.^29^ Similarly, in a large multicenter retrospective cohort of 1,045 liver transplant procedures, Martin-Mateos et al. identified prolonged hospital stay (>30 days), tracheal intubation exceeding 48 hours, and ICU stay longer than 72 hours as independent predictors of post-transplant MDR bacterial infections.^10^ Pre-transplant exposure also appears to play an important role. Tezcan et al., in a single-center study, demonstrated that pre-transplant hospitalization and ICU admission were significantly associated with the development of MDR infections after transplantation.^30^

Consistent with these observations, our study identified several variables showing significant positive associations with MDR infections on univariate analysis. Pre-transplant factors included prolonged hospitalization, ICU admission, and renal dysfunction. Post-operative factors associated with MDR infections were prolonged ICU stay (>7 days), mechanical ventilation exceeding 48-hour, requirement for hemodialysis or SLED, and postoperative complications such as intra-abdominal collections, pleural effusions, and vascular events. In addition, longer durations of invasive device use including Foley catheterization, arterial lines, central venous pressure lines, and abdominal drains as well as prolonged hospital stay, were significantly associated with MDR infections. The occurrence of CLABSI and intra-abdominal infections also demonstrated a strong association with MDR bacterial infections on univariate analysis.

To better characterize the independent determinants of MDR infections, we performed a phase-specific analysis encompassing pre-transplant variables, intraoperative events, postoperative factors, and overall hospital stay. This stratified analytical approach is consistent with recent systematic reviews and contemporary transplant-focused studies that emphasize the complex and multifactorial nature of infection risk across the transplant continuum.^20^,^31^

Our study identified CLABSI and intra-abdominal infections as independent risk factors for MDR infections within the first 30 days after LDLT, which suggest that MDR infections are multifactorial in origin. Brescini et al. evaluated the sources of bloodstream infections occurring within 30 days among 601 liver transplant recipients and observed that CVP lines were the most frequent source of bloodstream infection in their cohort, with high antimicrobial resistance rates among the isolates.^32^ Similarly, Chen et al. demonstrated that invasive devices and antimicrobial-resistant pathogens are major contributors to bloodstream infections after liver transplantation, many of which are caused by MDR organisms.^33^ Furthermore, Tezcan et al. reported a high frequency of intra-abdominal infections in the early post-transplant period and highlighted the substantial clinical burden of resistant organisms in the post–liver transplant infection landscape.^30^

Collectively, these findings indicate the need for a comprehensive prevention strategy to reduce MDR infections in liver transplant recipients. Such an approach should include routine screening for MDR colonization in pre-transplant setting, strict adherence to aseptic practices during surgery, early removal of invasive lines and drains, and empiric antibiotic therapy guided by local resistance patterns. Dolci et al. emphasized that incorporating colonization screening and early infection-control measures into transplant workflows can reduce MDR-related complications.^34^ Furthermore, the 2023 Infectious Diseases Society of America (IDSA) guidance by Tamma et al. provides updated recommendations for the treatment of antimicrobial-resistant gram-negative infections and antimicrobial stewardship programs applicable to transplant centers.^35^ Integrating antimicrobial stewardship into pre-transplant care is essential to limit unnecessary antibiotic exposure and help preserve the host microbiome, thereby reducing the risk of MDR organism colonization and infection.

To the best of our knowledge, this is among the first studies from Pakistan to systematically characterize the microbiological spectrum and perioperative risk predictors of MDR bacterial infections within 30 days of LDLT. Although published data from South Asian liver transplant centers on this specific topic remain scarce, the existing Pakistani literature on MDR organisms relates largely to general hospital settings rather than transplant programs.^15^,^16^ The high MDR rate of 49.7% observed here is consistent with the elevated gram-negative resistance burden prevalent across this region, which is a clinically important finding given the limited therapeutic options available locally. Unlike previous studies that primarily reported prevalence, this study evaluated risk factors across the entire transplant continuum and identified two potentially modifiable predictors: CLABSI and intra-abdominal infections. Both can be addressed through central-line bundles, early source control, and antimicrobial stewardship without relying solely on newer antimicrobial agents, making these findings particularly relevant in resource-limited transplant programs. The five years dataset, detailed microbiological analysis, and multivariable statistical approach are important strengths of this study.

### Limitations:

This study has limitations inherent to its retrospective, single-center design and its relatively short follow-up period of 30 days, which may not capture late-onset MDR infections or longer-term outcomes such as graft survival and mortality. Furthermore, because this was a single-center cohort study, the generalizability of these findings remains uncertain.

## CONCLUSION

MDR bacterial infections affected nearly half of all LDLT recipients within the first 30 days, with gram-negative organisms accounting for the majority of cases. Among all perioperative variables analyzed, CLABSI and intra-abdominal infections were the only independent predictors of MDR development. On the basis of these observations, we suggest the following recommendations:

- Implement standardized central-line care bundles in all liver transplant recipients.
- Establish protocols for early diagnosis and source control of intra-abdominal infections.
- Strengthen antimicrobial stewardship programs guided by local resistance patterns.

### Recommendations

Future studies should prospectively evaluate whether targeted prevention bundles reduce MDR infections and improve graft and patient outcomes.
